# Language patterns in Japanese patients with Alzheimer disease: A machine learning approach

**DOI:** 10.1111/pcn.13526

**Published:** 2023-02-08

**Authors:** Yuki Momota, Kuo‐ching Liang, Toshiro Horigome, Momoko Kitazawa, Yoko Eguchi, Akihiro Takamiya, Akiko Goto, Masaru Mimura, Taishiro Kishimoto

**Affiliations:** ^1^ Department of Neuropsychiatry Keio University School of Medicine Tokyo Japan; ^2^ Benesse Institute for Research on Continuing Care, Benesse Style Care Co., Ltd. Tokyo Japan; ^3^ Neuropsychiatry, Department of Neurosciences Leuven Brain Institute KU Leuven Belgium; ^4^ Tsurugaoka Garden Hospital Tokyo Japan; ^5^ Psychiatry Department Donald and Barbara Zucker School of Medicine New York New York USA

**Keywords:** Alzheimer disease, dementia, machine learning, natural language processing, speech‐language pathology

## Abstract

**Aim:**

The authors applied natural language processing and machine learning to explore the disease‐related language patterns that warrant objective measures for assessing language ability in Japanese patients with Alzheimer disease (AD), while most previous studies have used large publicly available data sets in Euro‐American languages.

**Methods:**

The authors obtained 276 speech samples from 42 patients with AD and 52 healthy controls, aged 50 years or older. A natural language processing library for Python was used, spaCy, with an add‐on library, GiNZA, which is a Japanese parser based on Universal Dependencies designed to facilitate multilingual parser development. The authors used eXtreme Gradient Boosting for our classification algorithm. Each unit of part‐of‐speech and dependency was tagged and counted to create features such as tag‐frequency and tag‐to‐tag transition‐frequency. Each feature's importance was computed during the 100‐fold repeated random subsampling validation and averaged.

**Results:**

The model resulted in an accuracy of 0.84 (SD = 0.06), and an area under the curve of 0.90 (SD = 0.03). Among the features that were important for such predictions, seven of the top 10 features were related to part‐of‐speech, while the remaining three were related to dependency. A box plot analysis demonstrated that the appearance rates of content words–related features were lower among the patients, whereas those with stagnation‐related features were higher.

**Conclusion:**

The current study demonstrated a promising level of accuracy for predicting AD and found the language patterns corresponding to the type of lexical‐semantic decline known as ‘empty speech’, which is regarded as a characteristic of AD.

Alzheimer disease (AD) is the leading cause of senile dementia, accounting for approximately half of all pathologically confirmed dementia cases.^1^, ^2^, ^3^, ^4^ The development of disease‐modifying therapeutics has increased the importance of accurate diagnosis and follow‐up evaluations.^5^ Although biomarker‐based examinations such as cerebrospinal fluid^6^, ^7^, ^8^, ^9^, ^10^ and amyloid and/or tau positron emission tomography^11^, ^12^ could be useful for such purposes, the need for specialized facilities, time, cost, or invasiveness of such examinations could make their use difficult in daily clinical settings.^13^, ^14^, ^15^ Blood biomarkers (e.g. plasma p‐tau) have also shown promise, but standard cutoff values have not yet been determined.^16^, ^17^

Even considering patient heterogeneity,^18^, ^19^, ^20^ patients with AD often present with language impairment as a major and distinctive symptom.^21^ Such impairments are characterized by less informative language production despite the use of a larger number of words, otherwise known as ‘empty speech’, and possibly reflect a lexical‐semantic decline.^22^, ^23^, ^24^, ^25^ Lesions in language‐related cortical and limbic structures might be relevant to these impairments.^20^, ^21^, ^26^, ^27^, ^28^

In this context, detailed analyses and assessments of language production might assist the diagnosis of AD and the follow‐up of disease progression.^18^, ^29^ Nicholas et al. (1985) investigated spoken discourse among patients with AD and described that a quantitative increase in deictic terms (e.g. this, that), pronouns, and semantic paraphasias and the repetition of the same statement formed ‘empty speech’; these findings were supported by subsequent studies.^18^, ^19^, ^29^, ^30^ Although such quantitative analyses suggest the presence of useful clinical markers, conventional analyses of discourse require somewhat labor‐intensive processes and examiner's judgments, which could cause evaluative inconsistencies.^31^

In this respect, current advances in computational approaches such as natural language processing (NLP) and machine learning are promising for their ability to improve objectivity and ease^19^, ^31^, ^32^ (for reviews, see^33^, ^34^, ^35^). NLP refers to a field of computer science concerned with computational techniques to learn, understand, and produce human language content.^36^ Machine learning applications use statistical algorithms, the performances of which improve with experience.^37^

Using an analysis that encompassed NLP, machine learning and factor analysis, Fraser et al. (2016) demonstrated an accuracy of over 81% for the prediction of AD based on spoken discourse during picture‐description tasks, identifying four distinctive factors: semantic impairment, acoustic abnormality, syntactic impairment, and information impairment; subsequent studies have indicated compatible findings using NLP^38^, ^39^ and machine learning.^32^, ^40^, ^41^

However, most previous studies have used large publicly available data sets^19^, ^32^ or relatively small local data sets (i.e. sample sizes of less than 30)^30^, ^42^, ^43^ in Euro‐American languages^32^ (for reviews, see^33^, ^34^, ^35^), while only a few studies with relatively small sample sizes have investigated the language patterns of Japanese‐speaking patients with AD.^38^, ^44^ Shimada et al. (1998) manually counted the number of elements that were described in a picture description task and reported that the amount of information conveyed and the efficiency of description were decreased in patients with AD (23 patients). Aramaki et al. (2016) used NLP and reported that vocabulary size (e.g. type‐token ratio and potential vocabulary size) could be an early indicator of cognitive impairment based on the comparison between healthy controls (HCs) and patients with mild cognitive impairment (MCI) (seven patients).

In addition, recent reviews have consistently pointed out that the various options of tools or methods for both NLP and machine learning make it difficult to compare or interpret findings among different studies of AD‐related language characteristics (for reviews, see^33^, ^34^, ^35^), whereas the improved accessibility for open‐source tools would enable better homogeneity of methods and reproducibility, even without advanced programming skills.^34^

In this study, therefore, we searched for AD‐related language patterns among Japanese patients with AD based on a relatively large data set, utilizing popular tools for a simple NLP pipeline and machine learning model. We speculated that such an exploration might benefit the development of objective measures for assessing language ability in patients with AD.

We obtained 276 speech samples (140 data sets from 42 patients with AD, 136 data sets from 52 HCs) using a picture description task. This task reportedly supports language production in patients with cognitive decline^45^ and is suitable for assessing lexical and semantic aspects,^46^ as well as practical and comprehensive language ability.^47^ While our analysis method was performed ‘by‐the‐numbers’ to enable an objective approach to identifying language patterns, as described below, the nature of this task also allowed a balancing of multiple, including practical, aspects of language.

To build a predictive model, we used part‐of‐speech and dependency as feature values that could be extracted from the speech data in a less arbitrary manner, and that could represent an elemental structure to understand the language patterns involving lexical, semantic, and grammatical aspects. To construct a single simple NLP pipeline, we used two popular tools: an open‐source NLP library for Python called spaCy,^48^ and an add‐on library called GiNZA, which is a Japanese parser based on Universal Dependencies.^49^, ^50^ Universal Dependencies has been developed as a project to enable cross‐linguistically consistent treebank annotation for many languages^49^, ^50^ and is now available in Japanese through GiNZA, thanks to the efforts of Japanese experts who adapted Japanese grammar and structure to the Universal Dependencies framework^51^, ^52^ (Fig. 1). For machine learning, we used eXtreme Gradient Boosting (XGBoost) as the classification algorithm. XGBoost is an algorithm that handles instance weights in an approximation of tree learning using a weighted quantile sketch procedure^53^; it enables to yield high prediction accuracy despite low computation time,^54^ and it has a built‐in capability to present feature importance, which improves the interpretability of the prediction.^53^ We also performed a box plot analysis to further understand the relationships between the features and the patient diagnosis and to interpret the predictions in a clinically reasonable manner.

**Fig. 1:**
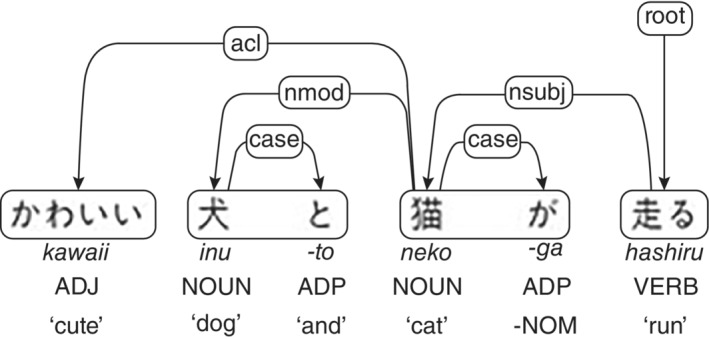
Japanese tagging for Universal Dependencies. ADJ, adjective; ADP, adposition; NOUN, noun; VERB, verb; acl, clausal modifier of noun (adjectival clause); case, case marking; nmod, nominal modifier; nsubj, nominal subject; root, root.

## Methods

### Participants

Participants were initially recruited from psychiatry departments at 10 different medical facilities in Japan, including Keio University Hospital, for the study PROMPT (Project for Objective Measures using Computational Psychiatry Technology), a prospective observational multicenter study aimed at developing objective severity evaluation technology for psychiatric disorders using voice, body motion, facial expressions, and daily activity data.^55^ The study was approved by the institutional review board of Keio University School of Medicine and registered with the University Hospital Medical Information Network (UMIN000021396 on https://www.umin.ac.jp/english/). HCs were also recruited and were examined using the Mini‐International Neuropsychiatric Interview (MINI)^56^ to confirm the absence of any psychiatric disorders. The recruitment period was from March 9, 2016, to March 31, 2019. Written informed consent was obtained from all participants. The participant's anonymity has been preserved. The authors assert that all procedures contributing to this work comply with the ethical standards of the relevant national and institutional committees on human experimentation and with the Helsinki of Declaration 1975, as revised in 2013. The analysis code in this study is available from the corresponding author upon reasonable request; however, the clinical data are unavailable because we did not receive consent from the participants to share their data publicly.

In this study, we used data from participants aged 50 years or older, and the patients with AD were diagnosed based on the National Institute on Aging‐Alzheimer's Association (NIA‐AA) criteria^21^ and did not present with obvious symptoms suggestive of primary progressive aphasia^57^, ^58^ or complications from other types of dementia, such as vascular dementia or dementia with Lewy bodies. We did not include patients with MCI because some of these patients would not necessarily progress to AD,^59^ and biomarkers for AD were not examined in this study. The average number of visits made by each participant were 3.89 times (SD = 1.88) with a minimum interval of 1 month, and the maximum data acquisition period was 23 months (mean of 8 months for the AD group and 8.5 months for the HC group). The demographic information of the participants is summarized in Table 1. To adjust for statistically significant group differences in age and education, we conducted a post hoc analysis on subpopulations (see Supporting information).

**Table 1:** Participant demographics and characteristics

Characteristic	Patients with AD	HCs
Data set	140	136
Participants (women)	42 (29)	52 (28)

	Patients with AD		HCs	
	Mean	SD	Mean	SD
Age	80.2	8.6	73.4	7.3*
Education, in years	12.2	3.2	14.3	2.9*
CDR	1.1	0.6	0.0	0.0*
MMSE	18.1	4.3	28.8	1.4*
Logical Memory II, WMS‐R	0.5	1.2	11.9	3.1*

*Note*: Age, education, and other test scores are values at baseline.

Abbreviations: AD, Alzheimer disease; CDR, Clinical Dementia Rating; HC, healthy control; MMSE, Mini‐Mental State Exam; SD, standard deviation; WMS‐R, Wechsler Memory Scale‐Revised.

* *p* < 0.05.

Each visit's data were labeled according to the criteria of the Alzheimer's Disease Neuroimaging Initiative 2 (ADNI 2)^60^ based on the Mini‐Mental State Examination (MMSE),^61^ the Logical Memory II from the Wechsler Memory Scale‐Revised (WMS‐R),^62^ and the Clinical Dementia Rating (CDR).^63^

The scores for the AD labels were MMSE ≤23, CDR ≥0.5, and Logical Memory II ≤8 (for ≥16 years of education), ≤4 (for 8–15 years), and ≤2 (for ≤7 years). The scores for the HC labels were MMSE ≥24, CDR 0, and Logical Memory II ≥9 (for ≥16 years of education), ≥5 (for 8–15 years), and ≥3 (for ≤7 years). Data that did not satisfy the criteria were excluded from the analysis.

### Data acquisition

MMSE, Logical Memory II, and CDR evaluations were conducted at each visit, and each participant had an approximately 10‐min semistructured conversation with the examiner. The conversation resembled a clinical interview performed in a relaxed mood, with questions about the participant's feelings, health, and daily life. Following this, the participant was also asked to complete a picture description task. In the task, the examiner showed the participant an illustration and said, ‘Please look at the picture and describe the scene depicted’. As a stimulus, we used a simple line drawing from the Visual Perception Test for Agnosia (VPTA).^64^ The theme of the drawing was ‘scapegoat’. It depicts a boy and two girls sitting laterally in a line, and in front of the middle girl, there is a dish with only one doughnut. The middle girl is visibly angry at the other girl sitting next to her because the middle girl believes the second girl ate one of the doughnuts. However, the boy sitting on the opposite side of the middle girl is shown to be eating the doughnut in question with some crumbs scattered in front of him and two doughnuts still on his dish. We chose this picture based on the ease of understanding for the examinees and to obtain speech samples containing various linguistic aspects (e.g. lexical, semantic, and syntactic). Since the picture description task would be more sensitive for lexical and semantic aspects, while the interview format would be sensitive for syntactic aspects,^46^ we estimated that the requirement to think about the undrawn background would bring the description close to that of the interview, and the speech sample would gain strength from both the picture description task and the interview format. During the task, if the examinee hesitated to speak or had trouble, the examiner would give brief responses to encourage the examinee. The task would end when the examiner judged that the examinee had answered as completely as possible. The recorded speech was manually transcribed by researchers who were trained in phonetic transcription, but they were not involved in the analysis in this study. All utterances, including filler, incomplete words, or misstatements (literal or verbal paraphasia) were transcribed, whereas phonetic characteristics such as pause length and prosodic contours were not annotated.

The different samples obtained from the same participant at each visit were not averaged because the multiple assessments for the same participant were performed at different times, and may therefore have different labels based on the cognitive test scores at the different assessment times (visits), which may possibly represent changes in cognitive abilities.

### Machine learning and statistical analysis

To train a computational classifier that can learn grammatical structure patterns to predict an AD diagnosis, we constructed predictive features based on part‐of‐speech and dependency information extracted from the speech contained in the picture description task data set. For each assessment, we processed the transcribed text using an open‐source NLP software library for Python called spaCy^48^, ^65^ using an add‐on library called GiNZA,^66^, ^67^ which is a Japanese parser based on Universal Dependencies.^49^, ^50^ Universal Dependencies is based on the annotation schemes of Stanford dependencies, in which grammatical relations are arranged in a hierarchy rooted with the most generic relation, in a dependent manner, and motivated by practical rather than theoretical concerns,^68^, ^69^ as well as other existing tag‐sets, with the goal of facilitating multilingual parser development.^49^, ^50^ Using spaCy and GiNZA, we extracted the part‐of‐speech and dependency tags of each word in an assessment. In total, we used 17 unique part‐of‐speech tags and 24 unique dependency tags.

After removing punctuations and stop‐words, we extracted the following two types of part‐of‐speech features in each assessment: (1) tag‐frequency features (i.e. frequencies/probabilities of appearance), and (2) tag‐transition features. To calculate the tag‐frequency feature values for an assessment, we counted the number of times each tag appeared in the participant's speech in the assessment and divided it by the total number of words. To calculate the tag‐transition feature values of an assessment, we collected all adjacent pairs of words in the participant's speech and denoted each tag pair as (Ti1, Ti2), 1 ≤ *i* ≤ *N*, where *N* is the total number of tag pairs in the assessment. In other words, the tag‐to‐tag transition probabilities can also be seen as the conditional probability of seeing tag *X*_*i*_ after seeing tag *X*_*i*−1_ in the previous word in the sentence. Such an approach has been commonly used, e.g. in pattern recognition for imaging,^70^ conditional random fields,^71^ and the hidden Markov model.^72^ For part‐of‐speech (17 unique tags), there were a total of 289 unique combinations of part‐of‐speech tag pairs (17^2^), and we counted the frequency of each unique combination in the *N* total pairs and divided by *N* to obtain the transition feature values of that unique tag‐to‐tag pair. We followed the above steps for dependency as well and obtained 24 dependency tag‐frequency feature values and 576 dependency tag‐transition feature values.

The tag‐frequency feature values and tag‐transition feature values of part‐of‐speech and dependency were used as input features to the XGBoost model,^53^ where we used a total of 906 features from both part‐of‐speech tags (17 tag‐frequency features and 289 tag‐transition features) and dependency tags (24 tag‐frequency features and 576 tag‐transition features).

Feature selection was performed as part of the XGBoost algorithm using a lasso‐like approach, reducing the probability of overfitting, while the total number of features (i.e. 906) was relatively large for the sample size (i.e. 276) and the number of participants (i.e. 94).

We selected the hyperparameters for the XGBoost model by performing a grid search, performed using a separate data set of speech samples from the same subject. This data set contains several questions and answers on different topics from the picture description task, comprising approximately the same number of utterances as the picture description task. Because the topics were different in the picture description task and the questions and answers, they did not contain highly related utterances that may bias the results (e.g. from information leakage). The set of hyperparameters with the highest average accuracy for the 100‐fold random cross‐validation was chosen to train an XGBoost model using the picture description task data set as follows: max_depth = 3, n_estimators = 700, learning_Rate = 0.1, subsample = 0.6, colsample_bytree = 1.0, gamma = 5, and min_child_weight = 1.

### Validation

To assess how well the models could be generalized with a low possibility of overfitting, we performed a 100‐fold repeated random subsampling validation.^73^ This method randomly samples data to build a training data set and uses the remaining samples for testing, then trains and tests the model multiple times, each time using a different randomly sampled data set, and uses the average of all of the trials as the final result. Our data set comprised 276 samples collected from 94 participants; not all participants had the same number of samples; we therefore used this validation method to balance the training data and ensure that samples from the same participants were either all in the training set or all in the validation set to avoid data leakage. In each fold, we randomly subsampled 90% of the participants and included all of the assessments of those participants as a training data set; the remaining 10% of the participants and all of their assessments were included in the test data set. Accordingly, data from the same participants (i.e. data from different visits made by the same person) were included either in the training data sets or the test data sets, but not in both.

In each fold of the repeated random subsampling validation, we extracted the feature importance values for each of the input features from the trained XGBoost model. The importance of a feature in the XGBoost model corresponded to the increase in the information gain based on Gini importance when splitting a tree at the corresponding feature within the tree; this parameter is related to how useful the features are in the construction of decision trees in a model that improves the overall predictive accuracy.^74^ The importance of each feature is averaged over the 100 models of the validation. The predictive accuracies, i.e. recall (sensitivity), specificity, precision, f1 scores (F‐measure: the harmonic mean of precision and recall), and area under the receiver operating characteristic curve (AUC), were calculated based on the classification of true‐positive, false‐positive, true‐negative, and false‐negative results. We also conducted the same analyses (i.e. predictive accuracy and feature importance) on a subpopulation matched for age and education between two groups as a post hoc analysis to consider the effect of significant differences in these demographics at baseline. The top 10 features with the highest averaged importance were selected for further analysis, such as qualitative considerations, as they were the most consistently important features in the 100‐fold validation. The statistical analyses utilized by machine learning were performed using the statistical package Statsmodels from Python, while the demographics analyses were performed using SPSS version 26.0 for Windows (IBM).

## Results

### Predictive accuracies

The model resulted in an f1 score (an overall measure of model accuracy) of 0.84 (SD = 0.06), a recall (i.e. sensitivity) of 0.84 (SD = 0.06), a specificity of 0.84 (SD = 0.09), a precision of 0.85 (SD = 0.06), and an AUC of 0.90 (SD = 0.03) (Table 2). The mean MMSE score of the patients with AD at baseline was 18.1 (SD = 4.3), while that at the final visit was 16.5 (SD = 4.9). The mean MMSE scores of the HC group were 28.8 (SD = 1.4) at baseline and 29.1 (SD = 1.0) at the final visit. In a post hoc analysis of a subpopulation matched for age and education (Table S1), the predictive accuracies did not change substantially (Table S2).

**Table 2:** Predictive accuracies

Performance measures	f1^†^	Precision^‡^	Recall^§^	Specificity^¶^	AUC
Mean	0.84	0.85	0.84	0.84	0.90
SD	0.06	0.06	0.06	0.09	0.03

Abbreviations: AUC, area under the curve; SD, standard deviation.

^†^ f1 = 2 × Precision × Recall/(Precision + Recall).

^‡^ Precision = TP / (TP + FP).

^§^ Recall (Sensitivity) = TP / (TP + FN).

^¶^ Specificity = TN / (FP + TN). TP, true positive; TN, true negative; FP, false positive; FN, false negative.

### Feature importance

Of the 906 features in total, in the trained XGBoost model, we found 120 features that had nonzero importance in at least one of the validations, which limited the possibility of overfitting (Table S3). Among the 10 most important features, seven were part‐of‐speech features: ‘noun followed by adposition’, ‘adposition followed by noun’, ‘verb’, ‘noun’, ‘symbol’, ‘particle’, and ‘interjection followed by particle’ (Table 3). The remaining three features were related to dependency, particularly dependency tag‐transitions: ‘determiner followed by coordinating conjunction’, ‘oblique nominal followed by nominal modifier’, and ‘numeric modifier followed by marker’. The importance values of the top three features were two or more times higher than those of the other features. In a post hoc analysis of a subpopulation matched for age and education, the results did not differ substantially (Table S4).

**Table 3:** Top 10 features in terms of importance as selected by XGBoost

Feature	Mean	SD
NOUN_ADP	31.5	3.0
ADP_NOUN	24.9	3.9
VERB	14.7	2.6
NOUN	8.5	1.2
SYM	8.4	1.2
det_cc	7.8	1.2
obl_nmod	7.1	1.4
PART	6.8	1.7
nummod_mark	6.5	0.8
INTJ_PART	6.5	0.4

*Note*: Capital letters represent part‐of‐speech tags. Small letters represent dependency tags. Single words represent tag features. Words connected by underbars represent tag transition features.

Abbreviations: ADP, adposition; cc, coordinating conjunction; det, determiner; INTJ, interjection; mark, marker; nmod, nominal modifier; NOUN, noun; nummod, numeric modifier; obl, oblique nominal; PART, particle; SYM, symbol; VERB, verb.

### Feature distributions

**Fig. 2:**
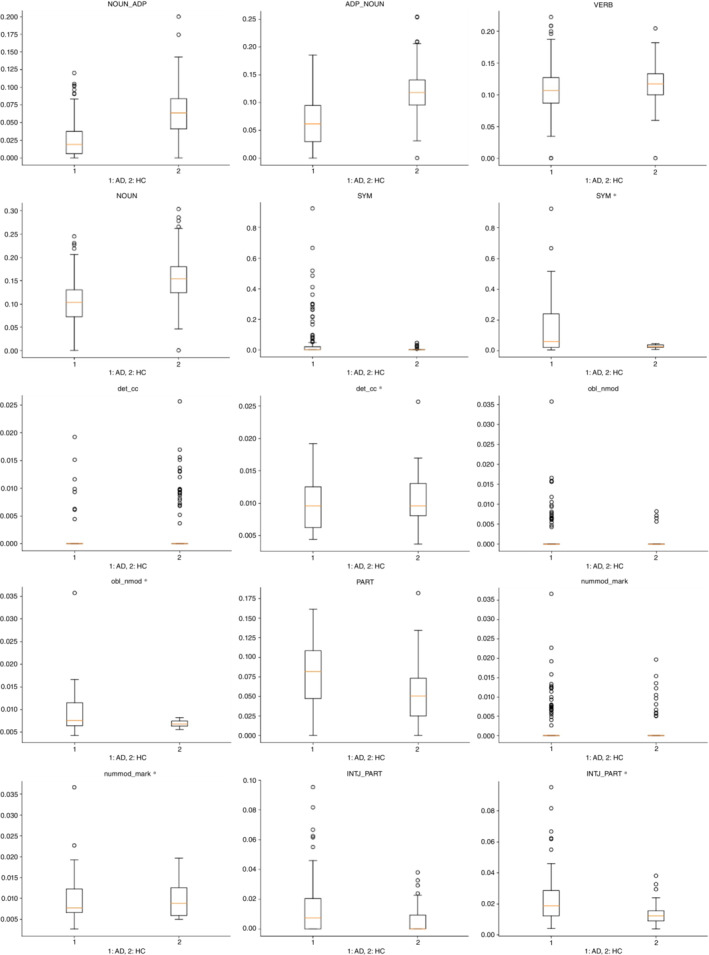
Distributions of the feature values for the top 10 features in terms of importance as extracted using XGBoost. * Zero‐values were eliminated to show the boxes clearly. Capital letters represent part‐of‐speech tags. Small letters represent dependency tags. Single words represent tag features. Words connected by underbars represent tag transition features. AD, patients with Alzheimer disease; ADP, adposition; cc, coordinating conjunction; det, determiner; HC; healthy controls; INTJ, interjection; mark, marker; nmod, nominal modifier; NOUN, noun; nummod, numeric modifier; obl, oblique nominal; PART, particle; SYM, symbol; VERB, verb.

Figure 2 illustrates the distributions of the feature values as box plots. Looking at the box plots, the frequencies (i.e. appearance rates) of ‘noun followed by adposition’, ‘adposition followed by noun’, ‘verb’, and ‘noun’ were lower in patients with AD, whereas those of ‘symbol’, ‘particle’, and ‘interjection followed by particle’ were higher. The differences in the frequencies of dependency‐related features, namely, ‘determiner followed by coordinating conjunction’, ‘oblique nominal followed by nominal modifier’, and ‘numeric modifier followed by marker’ were present but marginal since the differences between patients with AD and HCs were not visually obvious on the box plots.

## Discussion

In this study, 94 Japanese individuals (42 patients with AD and 52 HCs) completed a picture description task; the resulting 276 speech samples were then applied to a machine learning model. Using feature values based on part‐of‐speech and dependency, the model could predict AD with 84% accuracy and an AUC of 0.90. Among the features that were important for such predictions, seven of the top 10 features were related to part‐of‐speech (e.g. ‘noun followed by adposition’), while the remaining three were related to dependency (e.g. determiner followed by coordinating conjunction). Meanwhile, a box plot analysis demonstrated that the appearance rates of content words‐related features (e.g. ‘noun followed by adposition’) were lower among patients with AD, whereas those with stagnation‐related features (e.g. ‘symbols’) were higher. To our knowledge, these findings, which were obtained from a relatively large data set of Japanese speech using NLP and machine learning, are novel.

### Clinical implications

Our predictive model demonstrated an 84% accuracy, which is a promising level compared with previous studies,^19^, ^32^ and suggests the reliability of the feature values of the model.

Remarkably, seven of the top 10 important features determined using XGBoost and all top five features were related to part‐of‐speech, suggesting that part‐of‐speech–related features might be relatively more important than dependency‐related features for predicting language patterns in Japanese patients with AD.

More interestingly, the XGBoost model and the subsequent box plot analysis suggested that a decrease in content words–related features and an increase in stagnation‐related features, consistent with the components of ‘empty speech’ as described by Nicholas et al. (1985) and subsequent studies,^19^, ^30^, ^32^ were present.

The decrease in content words–related features in discourse is consistent with the findings of recent studies reporting that a lexical‐semantic decline is characteristic of AD,^38^, ^39^ including a machine learning study that demonstrated an over 81% accuracy using large data sets available from DementiaBank,^75^ a study identifying that semantic and information impairments were important factors,^19^ and a study demonstrating an 88% accuracy and reporting that a decrease in noun usage was an important factor.^43^

The increase in stagnation‐related features is also consistent with previous studies indicating that a period of silence is an important factor from an acoustic perspective in distinguishing patients with AD from HCs.^19^, ^39^, ^41^ In the present study, the higher appearance rates of ‘symbol’, ‘particle’, and ‘interjection followed by particle’ suggest an increase in stagnation and digression in the discourse of patients with AD. ‘Symbol’ is defined in Universal Dependencies as word‐like entities that differ from ordinary words in terms of form and function but are not punctuations, such as commas, periods, exclamation marks, or question marks.^76^ When we checked the transcription, most of them were represented by ‘…’, which indicates silence. ‘Particles’ covers functional words including particles or suffixes to add some nuances, and ‘interjection’ includes fillers such as ‘um’ and exclamations such as ‘oh’. Consequently, the frequent appearances of these features could be interpreted as stagnation related to word‐finding difficulty or utterances digressing from task‐consistent speech, such as the use of emotional words expressing the individual's impression of the picture.^32^

Another interesting finding was that three dependency‐related features based on adjacent tag‐to‐tag transitions (e.g. ‘nummod_mark’, i.e. a numeric modifier followed by a marker) were listed in the top 10 important features, whereas the group differences of the three features were not visually obvious in the box plots. Such diminutive differences in the dependency‐related features are consistent with a previous study, which indicated the absence of statistically significant differences in syntactic indices (e.g. average dependency distance) between Japanese patients with MCI and HCs.^38^

One implication is that nonlinear models (e.g. XGBoost) might enable the detection of subtle differences that are unlikely to be identified using linear models, while artificial intelligence approaches (e.g. machine learning) may lead to previously unappreciated findings and novel frameworks in a less biased, data‐driven manner.^77^

A possible interpretation of the marginal differences in the distributions of dependency‐related features between patients with AD and HCs observed in the box plot analysis would be that these features were indispensable for describing the task picture, in which three children are eating doughnuts. According to Universal Dependencies, a ‘determiner’ (det) represents deictic words (e.g. this, that), an ‘oblique nominal’ (obl) represents words used for temporal and locational nominal modifiers, and ‘marker’ (mark) represents subordinating conjunctions, conjunctive particles, or complementizers. Consequently, these features are likely necessary for describing the depicted scene, and likewise for ‘coordinating conjunction’ (cc) and ‘numeric modifier’ (nummod). Another interpretation of the marginal difference could be a linguistic peculiarity of Japanese, which allows a somewhat flexible word order.^52^, ^78^, ^79^ Consequently, the language patterns might have been somewhat obscured in terms of dependencies even though we used Universal Dependencies, which has been developed as a project to enable cross‐linguistically consistent treebank annotation for multiple languages.^49^, ^50^

The results might also be consistent with previous findings that syntactic and phonemic abilities may remain almost intact until later stages,^23^, ^27^, ^42^, ^80^ whereas lexical and semantic aspects would be impaired in early stages and decline further as the disease progresses.^22^, ^23^, ^24^, ^27^ Although this study is mostly based on patients who have moderate symptoms, some patients had mild to moderate symptom severity according to the mean MMSE score (i.e. 18.1 [SD = 4.3] at baseline), and these patients might not have presented with obvious syntactic impairment.^22^, ^23^, ^27^, ^42^

Overall, although the findings here might not translate directly to patients who have mild symptoms or who do not speak Japanese, they may provide a groundwork for future studies on language characteristics in patients with AD.

### Future directions

While the present study focused on a relatively simple NLP and machine learning pipeline, a comprehensive multivariate analysis would make future studies more robust. First, for semantic analysis, some outstanding techniques using a vector semantic approach have become popular.^81^, ^82^, ^83^ Vector semantics enable the representation of word meaning in NLP when examining spoken discourse,^84^ and they have recently been used to distinguish the features of speech uttered by patients with AD and those with MCI,^40^ similar to another line of research that we have been conducting. Second, as for pragmatic analysis, features such as word choice, voice tone or pauses could provide useful information in terms of the behavioral symptoms of AD, such as excuse behavior (e.g. head‐turning sign)^85^, ^86^ or ‘saving‐face’ behavior/responses.^87^, ^88^, ^89^ Last, in the context of a better understanding of the correlation between pathology and symptoms of AD, a combined analysis that incorporates biomarker‐based examinations of amyloid β or tau levels^90^ would be preferable.^21^, ^91^

### Limitations

This study had some limitations. First, there were statistically significant differences in age and education between the AD and HC groups. However, we conducted post hoc analyses on a subpopulation matched for age and education, and substantial group differences were not demonstrated (see Supporting information). Second, while our sample size of 276 data sets provided by 94 participants might be the largest used in a Japanese study to date^38^, ^41^ (for reviews, see^34^, ^92^), a larger sample might further increase the predictive accuracy and enable a deeper analysis, such as group comparisons according to disease stage. Third, a more complex stimulus, such as ‘The Cookie Theft’ used in the Boston Diagnostic Aphasia Examination,^93^ might improve data collection and analysis, since the stimulus picture used in the present study might be relatively simple for the purpose of obtaining a large number of words. Fourth, although the patients with AD in this study were clinically diagnosed based on examinations such as magnetic resonance imaging and single‐photon emission computerized tomography, other diseases (e.g. argyrophilic grain disease, frontotemporal lobar degeneration, and Lewy body disease) could not be ruled out completely.^1^, ^94^, ^95^, ^96^, ^97^ Likewise, as no biological or biophysical examinations were performed for the HCs, it is possible that these participants had asymptomatic diseases, including small vessel disease.^98^, ^99^, ^100^, ^101^, ^102^ Last, while Universal Dependencies has been designed to facilitate multilingual parser development^50^, ^51^, ^52^ and has recently become available for the Japanese language, some linguistic issues among different languages remain unsolved.^51^ Coping with these limitations and the further accumulation of research findings may refine the results and interpretations of future work.

## Conclusion

The current study demonstrated a promising level of accuracy for predicting AD and found the language patterns corresponding to the type of lexical‐semantic decline known as ‘empty speech’, which is regarded as a characteristic of AD. These findings would benefit the future assessment and diagnosis of AD.

## Disclosure statement

The authors declare no conflict of interest in relation to the submission of this manuscript. Within the past 3 years, Dr Horigome has received consultant fees from FRONTEO, Dr Eguchi has received speaker's honoraria from Eisai and Otsuka, Dr Takamiya has received grants or contracts from KAKENHI, SENSHINIYAKU, DAIWA SHOKEN, and Asteras, and speaker's honoraria from Otsuka and Dainippon Sumitomo, Dr Mimura has received grants and/or speaker's honoraria from Daiichi Sankyo, Dainippon‐Sumitomo Pharma, Eisai, Eli Lilly, FRONTEO, FUJIFILM RI Pharma, Janssen Pharmaceutical, Mochida Pharmaceutical, MSD, Nippon Chemipher, Novartis Pharma, Ono Pharma, Otsuka Pharmaceutical, Pfizer, Takeda Pharma, Tsumura, and Yoshitomi Pharma; Dr Kishimoto has received consultant fees from Dainippon Sumitomo, FRONTEO, KYOWA pharmaceutical industry, Novartis, and Otsuka, and speaker's honoraria from Banyu, Eli Lilly, Dainippon Sumitomo, Janssen, Novartis, Otsuka, and Pfizer. He has received grant support from Pfizer Health Research Foundation, Dainippon Sumitomo, Otsuka, and Mochida.

## Author contributions

Y.M. and K.L.: conceptualization, investigation, methodology, analysis, drafting the manuscript or figures; T.H.: conceptualization and editing the manuscript; M.K. and Y.E.: conceptualization and resources; A.T.: resources and editing the manuscript; A.G.: resources; M.M.: supervision and editing the manuscript; T.K.: supervision, funding acquisition, project administration, conceptualization, methodology, and editing the manuscript.

## Supporting information


**Table S1.** Participant demographics (post hoc)
**Table S2.** Predictive accuracies (post hoc)
**Table S3.** Features in terms of importance as selected by XGBoost
**Table S4.** Features in terms of importance as selected by XGBoost (post hoc)
