# Supplementary material for: Language patterns in Japanese patients with Alzheimer disease: A machine learning approach
Source: Psychiatry Clin Neurosci. 2023 Feb 8;77(5):273–81. doi: 10.1111/pcn.13526 (PMC11488616; doi:10.1111/pcn.13526)
Supplement: Supplementary file 1 — Table S1. Participant demographics (post hoc) Table S2. Predictive accuracies (post hoc) Table S3. Features in terms of importance as selected by XGBoost Table S4. Features in terms of importance as selected by XGBoost (post hoc) [file PCN-77-273-s001.docx]

Supplementary Table 1. Participant demographics (post-hoc)

|  | AD | | HC | |  |
| --- | --- | --- | --- | --- | --- |
| Data set | 113 | | 136 | |  |
| Participants (Women) | 31 (21) | | 52 (28) | |  |
|  | Mean | (SD) | Mean | (SD) |  |
| Age | 77.1 | (7.7) | 73.4 | (7.3) |  |
| Education, in years | 12.7 | (3.0) | 14.3 | (2.9) |  |
| CDR | 1.0 | (0.6) | 0.0 | (0.0) | * |
| MMSE | 17.8 | (4.7) | 28.8 | (1.2) | * |
| Logical memory Ⅱ (WMS-R) | 0.6 | (1.4) | 11.9 | (3.7) | * |

Age, education and other test scores are values at baseline. * P < 0.05; SD, standard deviation; CDR, Clinical Dementia Rating; MMSE, Mini Mental State Exam; WMS-R, Wechsler Memory Scale - Revised.

Supplementary Table 2. Predictive accuracies (post-hoc)

|  | f1^*1^ | Precision^*2^ | Recall^*3^ | Specificity^*4^ | AUC |
| --- | --- | --- | --- | --- | --- |
| Mean | 0.80 | 0.82 | 0.81 | 0.86 | 0.90 |
| (SD) | (0.08) | (0.08) | (0.08) | (0.10) | (0.07) |

AUC, area under the curve; SD, standard deviation. *^1^f1 = 2*Precision*Recall / (Precision + Recall); *^2^Precision = TP / (TP + FP); *^3^Recall (sensitivity) = TP / (TP + FN); *^4^Specificity = TN / (FP + TN). TP, true positive; TN, true negative; FP, false positive; FN, false negative.

Supplementary Table 3. Features in terms of importance as selected by XGBoost

|  | Mean | (SD) |
| --- | --- | --- |
| NOUN_ADP | 31.5 | (3.0) |
| ADP_NOUN | 24.9 | (3.9) |
| VERB | 14.7 | (2.6) |
| NOUN | 8.5 | (1.2) |
| SYM | 8.4 | (1.2) |
| det_cc | 7.8 | (1.2) |
| obl_nmod | 7.1 | (1.4) |
| PART | 6.8 | (1.7) |
| nummod_mark | 6.5 | (0.8) |
| INTJ_PART | 6.5 | (0.4) |
| INTJ | 6.4 | (0.4) |
| compound | 6.2 | (0.6) |
| VERB_ADP | 6.1 | (2.4) |
| ADP_PRON | 5.8 | (0.3) |
| NOUN_NUM | 5.6 | (3.1) |
| det_aux | 5.6 | (2.1) |
| AUX_VERB | 5.5 | (3.7) |
| cc | 5.1 | (5.2) |
| NOUN_VERB | 4.9 | (1.7) |
| DET_AUX | 4.8 | (3.2) |
| PRON | 4.8 | (2.5) |
| AUX | 4.7 | (2.6) |
| cc_fixed | 4.6 | (2.4) |
| nsubj | 4.6 | (3.9) |
| PART_VERB | 4.3 | (2.4) |
| NOUN_AUX | 4.2 | (2.8) |
| aux_mark | 3.8 | (3.2) |
| NUM | 3.7 | (3.8) |
| cc_mark | 3.6 | (3.1) |
| AUX_ADJ | 3.6 | (2.4) |
| ADJ_ADP | 3.4 | (2.8) |
| fixed_case | 3.3 | (2.7) |
| ADP_ADJ | 3.3 | (3.3) |
| SCONJ_AUX | 3.3 | (3.3) |
| nmod_det | 3.2 | (3.2) |
| nsubj_aux | 3.0 | (3.0) |
| INTJ_INTJ | 2.9 | (2.4) |
| nsubj_case | 2.8 | (2.9) |
| ADP_SYM | 2.8 | (4.3) |
| dep | 2.8 | (2.8) |
| NOUN_PRON | 2.8 | (3.5) |
| mark_advcl | 2.6 | (2.6) |
| nummod | 2.4 | (3.0) |
| det_mark | 2.4 | (3.0) |
| obl_nummod | 2.4 | (3.0) |
| compound_compound | 2.4 | (3.9) |
| ROOT | 2.3 | (2.9) |
| cop | 2.2 | (3.5) |
| advcl | 2.2 | (2.7) |
| ROOT_det | 2.2 | (2.6) |
| ADP_AUX | 2.0 | (2.5) |
| ADJ | 2.0 | (2.6) |
| case_mark | 1.9 | (2.9) |
| NOUN_PART | 1.8 | (2.8) |
| case_nmod | 1.8 | (2.2) |
| NUM_SCONJ | 1.6 | (2.5) |
| INTJ_SCONJ | 1.6 | (2.5) |
| aux_ROOT | 1.6 | (2.4) |
| fixed | 1.5 | (2.3) |
| mark_case | 1.4 | (2.9) |
| advmod_mark | 1.4 | (2.8) |
| PART_PART | 1.4 | (2.8) |
| DET_ADP | 1.2 | (2.5) |
| PART_AUX | 1.2 | (2.5) |
| ADP | 1.2 | (2.4) |
| mark_aux | 1.2 | (2.4) |
| ROOT_nummod | 1.2 | (2.3) |
| advmod | 1.2 | (2.3) |
| ROOT_nmod | 1.2 | (2.3) |
| SCONJ_VERB | 1.2 | (2.3) |
| NUM_ADP | 1.1 | (2.3) |
| NOUN_DET | 1.1 | (2.3) |
| PRON_PART | 1.1 | (2.2) |
| case | 1.1 | (2.2) |
| ROOT_case | 1.1 | (2.2) |
| aux | 1.1 | (2.1) |
| ADP_PART | 1.0 | (2.0) |
| obj | 1.0 | (1.9) |
| CCONJ_INTJ | 0.9 | (2.8) |
| PART_SCONJ | 0.9 | (2.7) |
| advcl_case | 0.9 | (2.6) |
| obl_case | 0.8 | (2.5) |
| SYM_SYM | 0.8 | (2.3) |
| CCONJ | 0.8 | (2.3) |
| ccomp_case | 0.7 | (2.2) |
| AUX_PART | 0.7 | (2.1) |
| DET_CCONJ | 0.7 | (2.1) |
| advcl_advcl | 0.7 | (2.0) |
| ADJ_NOUN | 0.6 | (1.9) |
| case_case | 0.6 | (1.9) |
| ADJ_ADJ | 0.6 | (1.9) |
| AUX_NOUN | 0.6 | (1.8) |
| nsubj_ROOT | 0.6 | (1.8) |
| ADV | 0.6 | (1.8) |
| case_obj | 0.6 | (1.8) |
| VERB_SCONJ | 0.6 | (1.8) |
| nsubj_mark | 0.6 | (1.8) |
| nmod_cc | 0.6 | (1.7) |
| ADP_DET | 0.6 | (1.7) |
| cop_acl | 0.6 | (1.7) |
| mark_mark | 0.6 | (1.7) |
| compound_case | 0.6 | (1.7) |
| INTJ_AUX | 0.6 | (1.7) |
| ADP_INTJ | 0.6 | (1.7) |
| CCONJ_ADP | 0.6 | (1.7) |
| acl_mark | 0.5 | (1.6) |
| ADJ_AUX | 0.5 | (1.6) |
| dislocated | 0.5 | (1.6) |
| nummod_ROOT | 0.5 | (1.5) |
| PRON_INTJ | 0.5 | (1.5) |
| mark | 0.5 | (1.5) |
| ADP_SCONJ | 0.5 | (1.5) |
| advcl_mark | 0.5 | (1.5) |
| case_ROOT | 0.5 | (1.5) |
| NUM_PART | 0.5 | (1.5) |
| aux_ccomp | 0.5 | (1.5) |
| ADV_SCONJ | 0.4 | (1.3) |
| nmod | 0.4 | (1.3) |
| det | 0.4 | (1.3) |
| DET_INTJ | 0.4 | (1.3) |

Features with zero values are not listed. SD, standard deviation. Capital letters represent part-of-speech tags. Small letters represent dependency tags. Single words represent tag feature values. Words connected by underbars represent tag transition feature values. ADJ, adjective; ADP, adposition; ADV, adverb; AUX, auxiliary; CCONJ, coordinating conjunction; DET, determiner; INTJ, interjection; NOUN, noun; NUM, numeral; PART, particle; PRON, pronoun; PROPN, proper noun; PUNCT, punctuation; SCONJ, subordinating conjunction; SYM, symbol; VERB, verb; X, other; acl, clausal modifier of noun (adjectival clause); advcl, adverbial clause modifier; advmod, adverbial modifier; amod, adjectival modifier; aux, auxiliary; case, case marking; cc, coordinating conjunction; ccomp, clausal complement; compound, compound; cop, copula; csubj, clausal subject; dep, unspecified dependency; det, determiner; discourse, discourse element; dislocated, dislocated elements; fixed, fixed multiword expression; mark, marker; nmod, nominal modifier; nsubj, nominal subject; nummod, numeric modifier; obj, object; obl, oblique nominal; punct, punctuation; root, root.

Supplementary Table 4. Features in terms of importance as selected by XGBoost (post-hoc)

|  | Mean | (SD) |
| --- | --- | --- |
| NOUN_ADP | 29.8 | (1.3) |
| ADP_NOUN | 25.9 | (3.2) |
| VERB | 13.8 | (1.9) |
| NOUN | 10.2 | (1.2) |
| SYM | 9.5 | (1.6) |
| INTJ | 7.4 | (1.2) |
| nummod_mark | 7.2 | (0.8) |
| AUX_VERB | 7.1 | (2.8) |
| PART | 7.0 | (0.8) |
| NOUN_VERB | 6.8 | (0.8) |
| obl_nmod | 6.8 | (4.0) |
| case_nmod | 6.2 | (3.4) |
| det_aux | 6.1 | (2.1) |
| compound | 5.6 | (0.7) |
| ADP_ADJ | 5.6 | (2.9) |
| cc | 5.6 | (6.0) |
| SCONJ_AUX | 5.4 | (2.9) |
| det_cc | 5.4 | (3.7) |
| AUX_ADJ | 5.2 | (1.9) |
| cc_mark | 5.1 | (3.4) |
| DET_AUX | 5.0 | (1.7) |
| INTJ_PART | 4.6 | (1.8) |
| PRON | 4.6 | (2.4) |
| VERB_ADP | 4.6 | (3.2) |
| nmod_det | 4.5 | (3.1) |
| ADP_PRON | 4.2 | (2.8) |
| ADP | 4.1 | (2.8) |
| INTJ_INTJ | 3.9 | (2.6) |
| CCONJ_ADP | 3.9 | (3.2) |
| nsubj_case | 3.6 | (3.1) |
| cc_fixed | 3.3 | (3.4) |
| obj | 3.3 | (2.7) |
| mark_aux | 3.3 | (3.4) |
| dep_ROOT | 3.1 | (3.1) |
| ADP_SYM | 2.9 | (4.5) |
| advcl_case | 2.8 | (2.9) |
| ADJ_ADP | 2.6 | (3.3) |
| mark_mark | 2.5 | (3.1) |
| nsubj_aux | 2.5 | (2.5) |
| cop | 2.5 | (3.1) |
| NOUN_AUX | 2.3 | (3.6) |
| PART_VERB | 2.3 | (2.9) |
| NUM | 2.1 | (3.2) |
| nsubj_mark | 2.0 | (2.6) |
| ADJ | 2.0 | (3.1) |
| case_obj | 1.9 | (2.4) |
| AUX | 1.9 | (2.9) |
| det_mark | 1.9 | (2.9) |
| mark_acl | 1.8 | (2.7) |
| ccomp | 1.7 | (2.7) |
| nsubj | 1.5 | (2.6) |
| aux_mark | 1.5 | (3.1) |
| SYM_SYM | 1.5 | (4.4) |
| NOUN_PART | 1.4 | (2.8) |
| NOUN_PRON | 1.4 | (2.8) |
| ADP_PART | 1.4 | (2.8) |
| nummod | 1.4 | (2.8) |
| NUM_SCONJ | 1.3 | (2.6) |
| PART_SCONJ | 1.3 | (2.6) |
| obl_nummod | 1.3 | (2.5) |
| fixed | 1.2 | (2.5) |
| advmod | 1.2 | (2.4) |
| ROOT_mark | 1.2 | (2.4) |
| aux | 1.1 | (2.3) |
| advcl_obl | 1.1 | (2.3) |
| ROOT_case | 1.1 | (2.2) |
| DET | 1.1 | (2.2) |
| VERB_INTJ | 1.1 | (2.2) |
| SCONJ | 1.1 | (2.2) |
| nummod_ROOT | 1.1 | (2.1) |
| ADP_DET | 1.0 | (2.0) |
| nsubj_ROOT | 1.0 | (2.9) |
| nmod_mark | 0.9 | (2.7) |
| DET_CCONJ | 0.9 | (2.6) |
| advmod_mark | 0.8 | (2.4) |
| ccomp_case | 0.8 | (2.3) |
| PART_AUX | 0.7 | (2.2) |
| dep | 0.7 | (2.0) |
| ADJ_PART | 0.7 | (2.0) |
| ROOT_nummod | 0.7 | (2.0) |
| acl_advmod | 0.7 | (2.0) |
| ROOT | 0.7 | (2.0) |
| ADV_PART | 0.7 | (2.0) |
| obl_mark | 0.6 | (1.9) |
| acl_case | 0.6 | (1.8) |
| aux_ROOT | 0.6 | (1.8) |
| SCONJ_VERB | 0.6 | (1.8) |
| DET_ADP | 0.6 | (1.8) |
| AUX_SCONJ | 0.6 | (1.7) |
| compound_aux | 0.6 | (1.7) |
| CCONJ_INTJ | 0.6 | (1.7) |
| case_case | 0.6 | (1.7) |
| ROOT_aux | 0.6 | (1.7) |
| fixed_case | 0.6 | (1.7) |
| SCONJ_ADJ | 0.5 | (1.6) |
| dep_dep | 0.5 | (1.6) |
| NOUN_NUM | 0.5 | (1.6) |
| NOUN_INTJ | 0.5 | (1.6) |
| aux_ccomp | 0.5 | (1.6) |
| nummod_discourse | 0.5 | (1.6) |
| compound_dep | 0.5 | (1.6) |
| advcl | 0.5 | (1.6) |
| advcl_mark | 0.5 | (1.5) |
| VERB_ADV | 0.5 | (1.5) |
| ADP_AUX | 0.5 | (1.5) |
| advcl_aux | 0.5 | (1.5) |
| ADP_INTJ | 0.5 | (1.5) |
| ADJ_ADJ | 0.5 | (1.5) |
| case | 0.5 | (1.5) |
| mark_case | 0.5 | (1.4) |
| NUM_PART | 0.4 | (1.3) |
| cop_acl | 0.4 | (1.2) |
| case_compound | 0.4 | (1.2) |
| obl_case | 0.4 | (1.2) |

Features with zero values are not listed. SD, standard deviation. Capital letters represent part-of-speech tags. Small letters represent dependency tags. Single words represent tag feature values. Words connected by underbars represent tag transition feature values. ADJ, adjective; ADP, adposition; ADV, adverb; AUX, auxiliary; CCONJ, coordinating conjunction; DET, determiner; INTJ, interjection; NOUN, noun; NUM, numeral; PART, particle; PRON, pronoun; PROPN, proper noun; PUNCT, punctuation; SCONJ, subordinating conjunction; SYM, symbol; VERB, verb; X, other; acl, clausal modifier of noun (adjectival clause); advcl, adverbial clause modifier; advmod, adverbial modifier; amod, adjectival modifier; aux, auxiliary; case, case marking; cc, coordinating conjunction; ccomp, clausal complement; compound, compound; cop, copula; csubj, clausal subject; dep, unspecified dependency; det, determiner; discourse, discourse element; dislocated, dislocated elements; fixed, fixed multiword expression; mark, marker; nmod, nominal modifier; nsubj, nominal subject; nummod, numeric modifier; obj, object; obl, oblique nominal; punct, punctuation; root, root.
